# Reliability of diagnostic coding in intensive care patients

**DOI:** 10.1186/cc6969

**Published:** 2008-07-29

**Authors:** Benoît Misset, Didier Nakache, Aurélien Vesin, Mickael Darmon, Maïté Garrouste-Orgeas, Bruno Mourvillier, Christophe Adrie, Sébastian Pease, Marie-Aliette Costa de Beauregard, Dany Goldgran-Toledano, Elisabeth Métais, Jean-François Timsit, The Outcomerea Database Investigators

**Affiliations:** 1 Intensive Care Unit, Fondation Hôpital Saint-Joseph, Université Paris-Descartes, Faculté de Médecine, 185 rue Losserand, 75014 Paris, France; 2 Conservatoire National des Arts et Métiers, 292 rue Saint Martin, 75003 Paris, France; 3 INSERM U823, Epidemiology of Cancer and Severe Illnesses, Albert Bonniot Institute, BP 217, 38043 Grenoble cedex 09, France; 4 Intensive Care Unit, Hôpital Saint Louis, Assistance Publique Hôpitaux de Paris, 1 avenue Vellefaux, 75010 Paris, France; 5 Intensive Care Unit, Fondation Hôpital Saint-Joseph, 185 rue Losserand, 75014 Paris, France; 6 Intensive Care Unit, Hôpital Bichat – Claude Bernard, Assistance Publique Hôpitaux de Paris, 48 rue Huchard, 75018 Paris, France; 7 Intensive Care Unit, Hôpital Delafontaine, Inserm EA 2511, Insitut Cochin, Paris, 2 rue Delafontaine, 93200 Saint Denis,, France; 8 Intensive Care Unit, Hôpital Beaujon, Assistance Publique Hôpitaux de Paris, 100 boulevard du Général Leclerc, 92118 Clichy cedex, France; 9 Intensive Care Unit, Hôpital Tenon, Assistance Publique Hôpitaux de Paris, 4 rue de la Chine, 75020 Paris, France; 10 Intensive Care Unit, Centre Hospitalier Général, 25 rue Pierre de Theilley BP 30071, 95503 Gonesse, France; 11 Intensive Care Unit, Hôpital Albert Michallon, Université Joseph Fourier, Faculté de Médecine, Grenoble, France

## Abstract

**Introduction:**

Administrative coding of medical diagnoses in intensive care unit (ICU) patients is mandatory in order to create databases for use in epidemiological and economic studies. We assessed the reliability of coding between different ICU physicians.

**Method:**

One hundred medical records selected randomly from 29,393 cases collected between 1998 and 2004 in the French multicenter Outcomerea ICU database were studied. Each record was sent to two senior physicians from independent ICUs who recoded the diagnoses using the *International Statistical Classification of Diseases and Related Health Problems: Tenth Revision *(ICD-10) after being trained according to guidelines developed by two French national intensive care medicine societies: the French Society of Intensive Care Medicine (SRLF) and the French Society of Anesthesiology and Intensive Care Medicine (SFAR). These codes were then compared with the original codes, which had been selected by the physician treating the patient. A specific comparison was done for the diagnoses of septicemia and shock (codes derived from A41 and R57, respectively).

**Results:**

The ICU physicians coded an average of 4.6 ± 3.0 (range 1 to 32) diagnoses per patient, with little agreement between the three coders. The primary diagnosis was matched by both external coders in 34% (95% confidence interval (CI) 25% to 43%) of cases, by only one in 35% (95% CI 26% to 44%) of cases, and by neither in 31% (95% CI 22% to 40%) of cases. Only 18% (95% CI 16% to 20%) of all codes were selected by all three coders. Similar results were obtained for the diagnoses of septicemia and/or shock.

**Conclusion:**

In a multicenter database designed primarily for epidemiological and cohort studies in ICU patients, the coding of medical diagnoses varied between different observers. This could limit the interpretation and validity of research and epidemiological programs using diagnoses as inclusion criteria.

## Introduction

Administrative coding of medical diagnoses has become mandatory in French hospitals in order to perform epidemiological studies and to calculate medical reimbursement costs. Most databases are used by hospital administrators, according to the local system for hospital funding, which is derived from the Diagnosis-Related Group (DRG) in the US [1]. In the French national system, the medical diagnoses are coded by the physician treating the patient, collected by the Department of Medical Information (DMI) in the hospital, and transmitted to a national service that determines the hospital costs to be reimbursed by the health care insurance system [2]. As in other countries [3,4], French intensive care unit (ICU) physicians have established a number of databases collating information from multiple centers in order to perform epidemiological studies and/or benchmarking [5]. The medical information in these databases, which share either a financial or a scientific objective, must be reliable. Most databases use a diagnostic thesaurus [6] extracted from the *International Statistical Classification of Diseases and Related Health Problems *(ICD) [7]. The 10th revision of this classification, the ICD-10, is used in France in the national funding database [2] and in the two main ICU databases used for clinical research [5,8]. The same revision is used in these databases to simplify data collection and comparisons.

In France, as in most Western countries, patients' medical records are now computerized in order to improve activity assessment. As diagnosis coding is a fastidious and time-consuming process, several groups have begun to develop automatic coding systems based on data available in hospital information systems [9]. However, preliminary results suggest that diagnosis coding in economic databases is inconsistent between physicians and administrative personnel [10,11].

The Outcomerea database was set up in 1998 in order to perform clinical research on ICU cohorts. It contains a pre-established set of physiological data, clinical diagnoses, and therapeutic procedures collected every day during a patient's ICU stay. It receives data from 12 French ICUs [5]. Each year, the participating ICUs must collect data during the complete ICU stay of at least 50 patients staying for more than two consecutive days. Good reliability of physiological data designed to calculate severity scores has been documented following biannual audits [12]. The diagnoses are coded according to the guidelines published by the French Society of Intensive Care Medicine (SRLF) and the French Society of Anesthesiology and Intensive Care Medicine (SFAR) in 1999 [13]. Large cohorts based on coded diagnoses are regularly published and used to document epidemiological trends and the outcome of acute diseases such as sepsis [14-16]. However, the results of these studies are regularly challenged [17].

Our hypothesis was that the poor reproducibility of medical diagnoses observed in administrative databases is also found in research databases. The present study tested the reliability of coding of medical diagnoses, and specifically the diagnoses of septicemia and hemodynamic shock, in the Outcomerea database.

## Materials and methods

### Database and intensive care units

This study was performed in the 12 ICUs providing data for the Outcomerea database [5]. The quality of this database has been confirmed by periodic auditing [12,18] of the administrative and physiological data and of severity scores. The contact physicians for the database in the participating ICUs are listed in Additional file 1 and have been accredited for intensive care practice according to French law [19].

### Data source: medical records

In each ICU, the physician treating the patient elaborates a medical record describing the ICU stay and codes the diagnoses for both funding and Outcomerea databases. The aim of the record is to transmit information to the corresponding specialist and/or the patient's general practitioner. The structure of the database was predefined separately in all units. Its content includes the reason for ICU admission, prior diagnoses or comorbidities, a summary of events leading to admission, clinical and paraclinical details noted at admission and over the course of the ICU stay, treatment at discharge, and a conclusion summarizing the stay. The record is comprised of 1,000 to 2,000 words, representing two to three typed pages.

### Diagnosis coding

Coding is performed using the ICD-10 during the ICU stay and immediately at the time of ICU discharge and medical record writing. The treating physician allocates only one set of codes per patient. Coding concerned only data from the ICU stay since stays on other wards are assessed by the ward physicians. It includes a principal diagnosis, which plays a central role in the group allocation in the funding database [2]. The choice of the principal diagnosis follows SRLF/SFAR guidelines [13]. The ICD-10 includes around 52,000 codes [7]. Each code consists of a letter followed by a number with at least two digits. The ICD-10 arborescence allows us to increase the details of the code by adding a digit to 'father' codes. For instance, diseases of the genital and urinary system begin with the letter 'N', the first three digits of the acute renal failure code are N17, and the fourth digit determines the mechanism of acute renal failure (tubular necrosis: N170, cortical necrosis: N171, and so on). Of the 662 codes proposed by the SRLF/SFAR guidelines [13], 49 (7%), 559 (84%), and 54 (8%) consist of three, four, or more than four digits, respectively. Agreement testing was performed after truncating to four those codes that consist of more than four digits. We did not assess the reliability of the therapeutic codes.

One hundred medical records were selected randomly from 29,393 cases collected in the database between 1998 and 2004 using SAS software (SAS Institute Inc., Cary, NC, USA). The selection was balanced between hospitals. The original diagnostic codes selected by the physician treating the patient for DRG allocation were obtained from the DMI physician of each hospital. This physician was required to code in accordance with SRLF/SFAR guidelines [13] but did not have to follow specific regular training. Each record was sent to two senior investigators from the Outcomerea database; these physicians worked in two ICUs (which were independent from the ICU caring for the patient) and were blinded to the original coding. Both physicians had received specific training in accordance with SRLF/SFAR guidelines [13] during a 3-hour session at implementation of the database and then every 2 years or on recruitment of a new coder in each center. The coding of their first 10 records was audited.

Both investigators were asked to allocate a new diagnosis code after carefully reading each medical record. Thus, three independent series of codes were obtained per patient including the initial coding provided by the physician treating the patient. A specific subanalysis was performed in patients for whom one of the three coders had selected a code derived from R57 (hemodynamic shock) or A41 (septicemia). The truncation of these codes is symbolized as R57- and A41-.

The allocation of the codes was compared between the three coders, independently of the code's ranking in a single patient. For example, if 'sepsis' was coded first by one physician and coded second by another, the two physicians were considered to agree. The results are expressed as mean ± standard deviation (SD) or 95% confidence interval (95% CI) as appropriate. Differences between selected codes are described qualitatively. The reliability between the coders was assessed by kappa statistics for multiple raters [20]. The interpretations of the kappa values are as follows: 0.00 = no agreement, 0.01 to 0.20 = slight agreement, 0.21 to 0.40 = fair agreement, 0.41 to 0.60 = moderate agreement, 0.61 to 0.80 = substantial agreement, and 0.81 to 1.00 = almost perfect agreement.

### Ethical issues

According to French law, this study did not require the consent of patients as it involved research on the quality of a database collection. The study was accordingly approved by the institutional review board of the Groupe Hospitalier Paris Saint-Joseph.

## Results

### Number of diagnosis codes per patient

**Figure 1:**
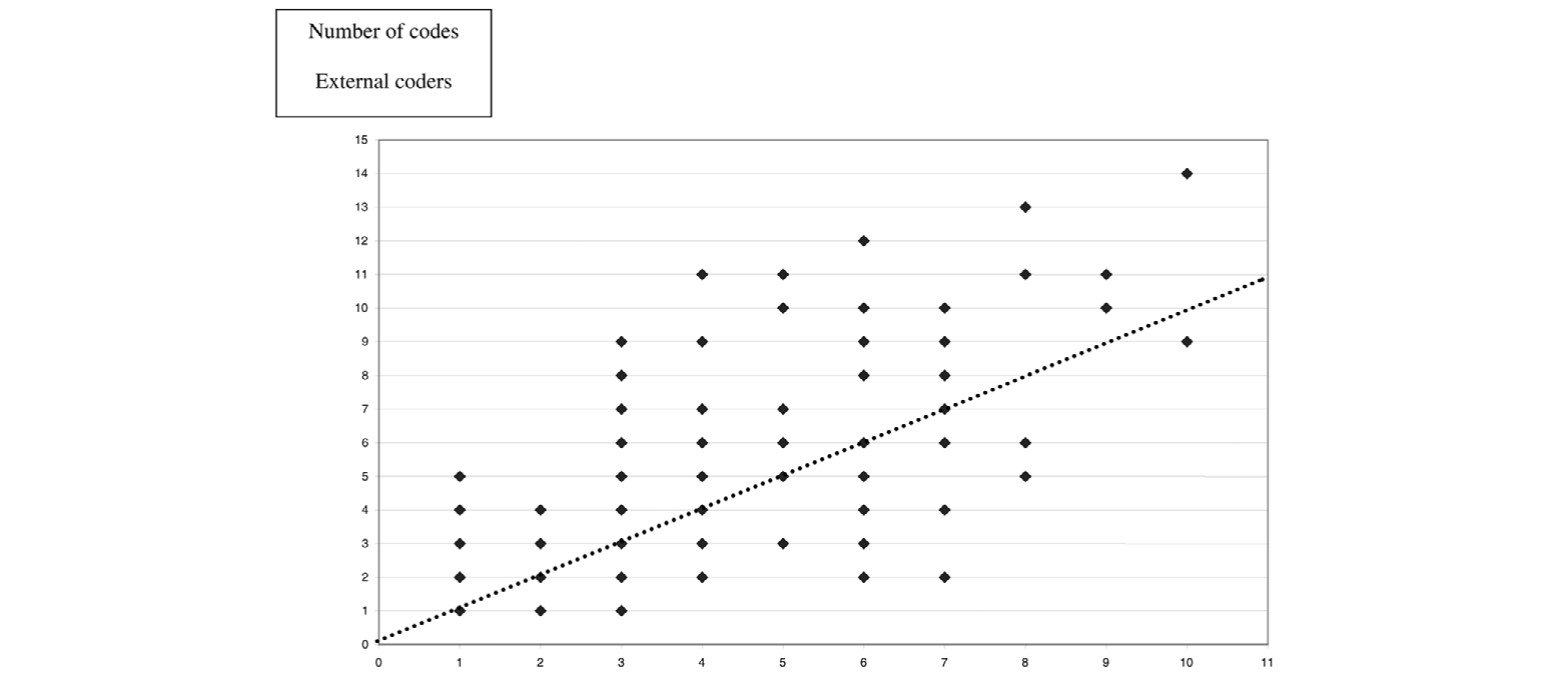
Number of codes per patient selected by the initial coder (x-axis) and the two external coders (y-axis). The dotted line represents identity.

The physicians coded an average (± SD) of 4.6 ± 3.0 (median 5, range 1 to 32) diagnoses per patient in the 29,393 cases in the Outcomerea database. The investigators coded a total of 1,389 diagnoses for the 100 selected patients. There was no significant difference in the average number of codes selected by the original physician and the two external coding physicians: 4.12 ± 2.26, 5.46 ± 3.22, and 4.31 ± 2.14, respectively (*P *> 0.20). Figure 1 shows a large scatter between initial coding and external coding, irrespective of the initial count.

### Qualitative data

The 11 most common diagnoses were acute respiratory failure (J960, n = 78); bacterial pneumonia, unspecified (J159, n = 31); essential hypertension (I10, n = 25); left ventricular failure (I501, n = 22); coma, unspecified (R402, n = 21); chronic renal failure, unspecified (N189, n = 21); cardiogenic shock (R570, n = 21); gastrointestinal hemorrhage, unspecified (K922, n = 17); convulsions, other and unspecified (R568, n = 6); other shock (R578, n = 16); and septicemia, unspecified (A419, n = 16).

**Figure 2:**
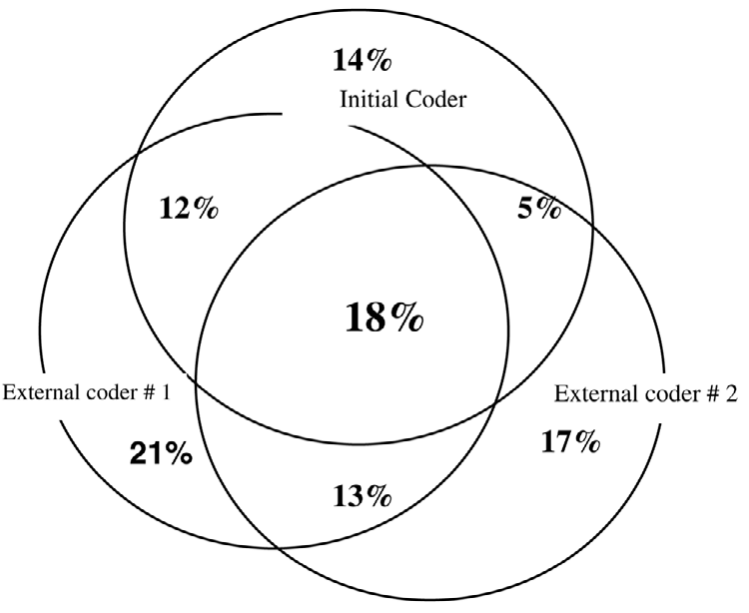
Distribution of codes according to the three coders. Each coding is symbolized by a circle. Only 18% of the codes (intersection of the three circles) were selected by all three coders.

The main diagnosis used for the DRG system by the initial physician was matched by both external coders in 34% (95% CI 25% to 43%) of patients, by only one in 35% (95% CI 26% to 44%) of patients, and by neither in 31% (95% CI 22% to 40%) of patients. The proportion of all codes (that is, not just the main diagnoses) which were selected by the initial physician and by at least one of the two external coders varied between 25% (95% CI 21% to 29%) and 60% (95% CI 55% to 65%). The variability in number of initial diagnoses explained only 63.6% of the variability in diagnoses selected by the two external coders (*P *< 0.0001). Figure 2 shows the proportion of codes, which were selected by one, two, or all three coders: 52% (95% CI 49% to 55%) were selected by one, 30% (95% CI 28% to 32%) by two, and only 18% (95% CI 16% to 20%) by all three coders.

**Figure 3:**
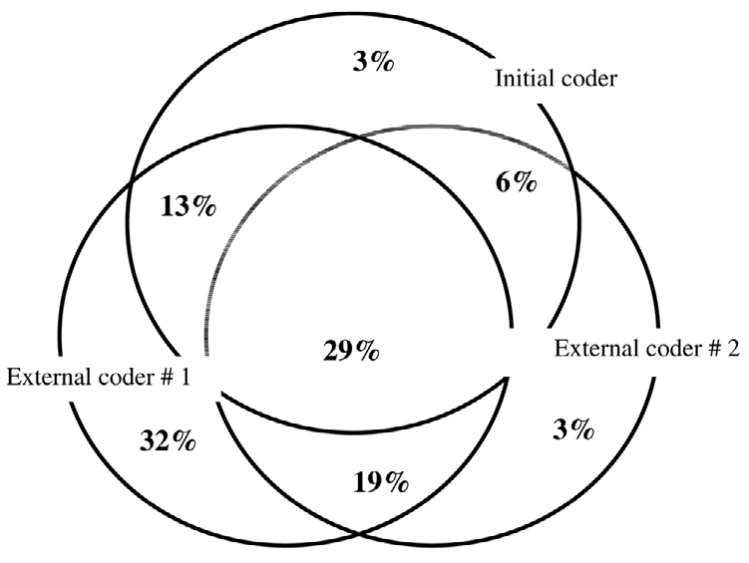
Distribution of the codes for shock (beginning with R57) according to the three coders. Each coding is symbolized by a circle. Only 29% of the codes (intersection of the three circles) were selected by all three coders.

The kappa statistics performed for the four most frequent codes indicate moderate agreement between the initial and external coders (Table 1). A substantial agreement was observed only between the two external coders for two codes (R402 and I501) (Table 2). A diagnosis of septicemia (A41-) or shock (R57-) was coded by the original physician in 8 (8% [95% CI 3% to 13%]) and 15 (15% [95% CI 8% to 22%]) patients, by all three coders in 6 (6% [95% CI 1% to 11%]) and 9 (9% [95% CI 3% to 15%]) patients, and by at least one coder in 15 (15% [95% CI 8% to 22%]) and 31 (31% [95% CI 22% to 40%]) patients, respectively (see Figure 3 for shock). The kappa statistics performed for the 'father' codes of septicemia (A41-) and shock (R57-) indicate moderate to substantial agreement between the three coders (Table 3). Finally, the kappa coefficient between the three coders was 0.26 (95% CI 0.14 to 0.38), indicating poor agreement.

**Table 1:** Agreement between the initial and each external coder for the four most frequently selected diagnoses

		Initial versus external coder 1		Initial versus external coder 2	
	Number	Kappa	95% CI	Kappa	95% CI

J960, acute respiratory failure	78	0.26	0.06–0.46	0.25	0.06–0.43
J159, bacterial pneumonia, unspecified	31	0.49	0.22–0.76	0.26	0.03–0.56
I10, essential hypertension	25	0.52	0.25–0.79	0.26	0.06–0.59
I501, left ventricular failure	22	0.50	0.18–0.81	0.46	0.10–0.82

CI, confidence interval.

**Table 2:** Agreement between the two external coders for the most frequently selected diagnoses

	Number	Kappa	95% CI
J960, acute respiratory failure	63	0.42	0.23–0.61
J159, bacterial pneumonia, unspecified	22	0.49	0.22–0.76
R402, coma, unspecified	21	0.82	0.63–1.00
I501, left ventricular failure	17	0.67	0.42–0.94

CI, confidence interval.

**Table 3:** Agreement between the three coders for the 'father' codes of septicemia and shock

	Initial versus external coder 1		Initial versus external coder 2		Between the two external coders	
	Kappa	95% CI	Kappa	95% CI	Kappa	95% CI

A41-, septicemia	0.69	0.47–0.92	0.71	0.47–0.94	0.77	0.58–0.96
R57-, shock	0.60	0.38–0.81	0.51	0.31–0.70	0.55	0.36–0.74
A41- or R57-	0.70	0.54–0.86	0.49	0.53–0.85	0.73	0.58–0.87

CI, confidence interval.

## Discussion

In this study investigating the reliability of diagnostic coding by physicians trained to collect data in ICU patients, we observed that coding by an external physician after examination of a patient's medical record did not modify the total number of diagnoses made for the patient. Agreement between coders was most often moderate regarding the choice of codes. This was also true for the principal diagnosis used for the DRG system as well as for the codes used to indicate septicemia and shock.

Hospital databases are used to estimate reimbursement costs of medical care, to determine human resources for clinical units, or to perform epidemiological studies. Accurate coding of diagnoses is a cornerstone of these three objectives. Quality analyses of coding have been performed mainly in the area of resource allocation. At the hospital level, these analyses have shown that coding is poorly reliable. It has been estimated that external coding in European countries and the US would modify 32% to 42% of diagnoses [10]. The quality control system of Medicare showed that reliability was as poor between external coders as between physicians and hospital administrators [11]. Finally, the use of trained experts to carry out coding increases the number of diagnoses but the level of agreement between experts is less than 70%. In American ICUs, the codes describe the reason for admission in less than 50% of cases, devaluing hospitals with ICUs and making the administrative database nonapplicable for quality-of-care assessment [21].

Coding reliability appears to be even worse in medical ICUs. In ICU patients, coding errors concern as many as 46% of cases, with a resultant financial loss of 18.4% [22]. Coding of therapeutic procedures plays an important role in most systems derived from North-American DRGs. This accounts for the better accuracy of DRGs in elective surgical patients [23]. Accordingly, in contrast to diagnostic coding, the French network CUB-Rea of 35 ICUs around Paris showed that the reliability of coding of severity scores and therapeutic items was acceptable [8]. The poor reliability we found for diagnoses could be due to the frequent combination of multiple diseases and organ failure in a single patient, which plays a cumulative role in resource utilization, mortality, and secondary morbidity [24]. ICD codes are often used in large epidemiological studies as a surrogate for the cause of ICU admission [14-16]. However, the use of such codes in classifying ICU patients has been widely debated and other tools for classifying ICU admissions have been proposed [3,25]. Thus, coding requires complex and precise rules [13], especially in the ICU setting, to select diagnoses with objectivity. This can be obtained through an automated algorithm using an expert system [9]. We have recently designed software that selects the codes from the patient's electronic record, based on linguistic treatment exploring inductive mechanisms and extracting concepts rather than words from textual medical reports [26]. Testing of this software is currently under way in a pilot cohort of patients [26].

We chose to perform this study with real data from patients admitted to ICUs corresponding to French quality standards [19] and sharing a routine practice in database exploitation [5]. External coding was performed by two independent experts who had been trained in coding in a similar way and had similar experience in ICU practice.

Despite these precautions, our study has several limitations due to the small sample size, the methods used, and the fact that codes were determined by physicians rather than trained administrative coders. First, external coding was performed after the ICU stay by practitioners following a *post hoc *chart review. It is more likely that the initial diagnosis made by the physician treating the patient was accurate and that the chart review may not have correctly captured the appropriate diagnosis and is therefore inaccurate. This could also account for the poor reliability between the two external coders. This suggests that neither a gold standard nor an expertise for diagnosis coding exists in the ICU. Second, the external coders worked in hospitals with different case mixes and could have had different areas of scientific interest. Thus, their method of coding could have been influenced by their professional expertise. We attempted to control for this factor by training them to code according to specific guidelines. However, these guidelines, even if they should be considered as the French 'gold standard', include the 662 codes considered to be the most common, and this number might be too large to use with good reliability. Third, we did not control the quality of the medical records, corresponding to 'real-life' recording in France. However, all the summaries corresponded to the quality criteria required for French hospital certification procedures [27]. Again, this does not account for the poor reliability between the external coders as they worked on the same source documents. Finally, the reliability of coding septicemia and shock requires further assessment, particularly to optimally interpret both previous and future cohort studies using administrative data.

## Conclusion

Using a quality-assured database designed for clinical research, we observed that coding of medical diagnoses was unreliable in ICU patients despite specific training of physicians. From an economic point of view, this could explain the poor results of the DRG system in ICU patients which have been previously published. This lack of reliability could limit the interpretation of epidemiological and clinical research programs based on diagnoses such as sepsis. The reliability of diagnoses should be tested in other research databases, and systems of automatic computerized data collection [9] should be analyzed. The results of our study will be used as a comparator in a forthcoming investigation of automatic coding in ICU patients.

## Key messages

• Coding diagnoses is necessary to categorize patients in epidemiological studies.

• Multiple symptoms or diseases are characteristic of intensive care unit (ICU) patients.

• The *International Statistical Classification of Diseases and Related Health Problems *provides a profusion of medical codes.

• The selection of codes by ICU physicians is unreliable. This weakens the conclusions of cohort studies using diagnosis as an inclusion criterion.

## Abbreviations

DMI = Department of Medical Information; DRG = Diagnosis-Related Group; ICD = *International Statistical Classification of Diseases and Related Health Problems*; ICD-10 = *International Statistical Classification of Diseases and Related Health Problems: Tenth Revision*; ICU = intensive care unit; SD = standard deviation; SFAR = French Society of Anesthesiology and Intensive Care Medicine; SRLF = French Society of Intensive Care Medicine.

## Competing interests

The authors declare that they have no competing interests.

## Authors' contributions

BM, DN, and J-FT participated in the conception and design of the study and in the writing of the article. AV participated in the writing of the article. All of the authors participated in the acquisition of data, analysis and interpretation of data, critical revision of the manuscript for intellectual content, and approval of version to be published. All authors read and approved the final manuscript.
